# Inhibition of the mechanistic target of rapamycin induces cell survival via MAPK in tuberous sclerosis complex

**DOI:** 10.1186/s13023-020-01490-w

**Published:** 2020-08-17

**Authors:** Yiyang Lu, Erik Y. Zhang, Jie Liu, Jane J. Yu

**Affiliations:** 1 Department of Internal Medicine, Pulmonary, Critical Care and Sleep Medicine, University of Cincinnati College of Medicine, 231 Albert Sabin Way-ML 0564, Cincinnati, OH 45267 USA; 2 Department of Pulmonary and Critical Care Medicine, Guangzhou Institute for Respiratory Health, State Key Laboratory of Respiratory Disease, National Clinical Research Center for Respiratory Disease, The First Affiliated Hospital of Guangzhou Medical University, Guangzhou, Guangdong China

**Keywords:** Tuberous sclerosis complex, Lymangioleiomyomatosis, Tumor progression, MAPK signaling pathway, Rapamycin, Cell survival

## Abstract

**Background:**

Tuberous sclerosis complex (TSC) is a genetic disorder that cause tumors to form in many organs. These lesions may lead to epilepsy, autism, developmental delay, renal, and pulmonary failure. Loss of function mutations in *TSC1* and *TSC2* genes by aberrant activation of the mechanistic target of rapamycin (mTORC1) signaling pathway are the known causes of TSC. Therefore, targeting mTORC1 becomes a most available therapeutic strategy for TSC. Although mTORC1 inhibitor rapamycin and Rapalogs have demonstrated exciting results in the recent clinical trials, however, tumors rebound and upon the discontinuation of the mTORC1 inhibition. Thus, understanding the underlying molecular mechanisms responsible for rapamycin-induced cell survival becomes an urgent need. Identification of additional molecular targets and development more effective remission-inducing therapeutic strategies are necessary for TSC patients.

**Results:**

We have discovered an Mitogen-activated protein kinase (MAPK)-evoked positive feedback loop that dampens the efficacy of mTORC1 inhibition. Mechanistically, mTORC1 inhibition increased MEK1-dependent activation of MAPK in TSC-deficient cells. Pharmacological inhibition of MAPK abrogated this feedback loop activation. Importantly, the combinatorial inhibition of mTORC1 and MAPK induces the death of TSC2-deficient cells.

**Conclusions:**

Our results provide a rationale for dual targeting of mTORC1 and MAPK pathways in TSC and other mTORC1 hyperactive neoplasm.

## Background

Tuberous sclerosis complex (TSC) is a genetic disorder that is associated with tumors to form in many organs, primarily in the brain, eyes, heart, kidney, skin and lungs [1]. These lesions cause morbidity and mortality in patients with TSC, as they may lead to epilepsy, autism, developmental delay, renal, and pulmonary failure [2]. Loss of function mutations in *TSC1* and *TSC2* genes are the known causes of TSC. The TSC1 and TSC2 gene products combine with TBC1D7 to form a ternary complex which have GTPase activating protein (GAP) activity for the GTPase Ras homologue enriched in brain (Rheb), therefore inhibiting mTOR complex 1 (mTORC1) kinase activity [3, 4]. Therefore, Targeting mTORC1 becomes a most available therapeutic strategy for TSC.

The mechanistic target of rapamycin (mTOR) is a serine/threonine protein kinase that regulates cell growth, proliferation, cell motility, cell survival, protein synthesis, autophagy, and transcription [5]. The mTOR functions as a catalytic subunit in two distinct multiprotein complexes, mTORC1 and mTORC2 [6]. mTORC1, a complex including regulatory-associated protein of mTOR (RAPTOR), phosphorylates and controls, at least, two regulators of protein synthesis, the 40S ribosomal protein subunit S6 kinase (S6K) and the translational repressor 4E-binding protein 1, referred as 4E-BP1. mTORC2, characterized by rapamycin-insensitive companion of mTOR (RICTOR), phosphorylates several AGC protein kinases, including AKT at Ser473. Deregulation of mTORC1 has been observed with various human diseases [7]. Thus, this renders mTORC1 as an attractive drug target for cancer therapy. Although mTORC1 inhibitors showed very convincing results in some TSC clinical studies, tumors or lung function returned to their original states when drugs were discontinued, addressing the cytostatic instead of cytotoxic effects of mTORC1 inhibition [8–10]. Thus, there is an urgent need to identify additional molecular targets and develop novel combinatorial therapies with mTORC1 inhibitors that could render tumor cell death.

To explore the possibility of selectively killing tumor cells with high mTORC1 activity, we performed bioinformatic analysis and identified signaling pathways that were activated in response to rapamycin treatment, including focal adhesion, adherent junction, Jak-Stat, and MAPK signaling pathways. Recently, the FAK inhibitor and JAK-STAT inhibitor have shown benefits in mTORC1 inhibitor-resistant pancreatic cancer and breast cancer, respectively [11, 12]. MAPK inhibitors have been studied with a synergistic effect with mTOR inhibitors in several cancers [13, 14]. However, the mechanism of MAPK inhibitor-attenuated resistance to mTORC1 inhibition in cancers and especially in TSC have not been extensively explored.

Here we report that mTORC1 inhibition results in a compensatory activation of MAPK signaling pathway in TSC-deficient cells in vitro. This enhanced MPAK signaling pathway was associated with enhanced survival of TSC-deficient cells. Pharmacological suppression of MEK1/2-MAPK sensitized TSC-deficient cells to cell death. Taken together, our study reveals a novel approach of combined suppression of pro-survival signaling pathways that informs future preclinical studies and potential clinical application of remission-inducing therapies for TSC and other mTOR1 hyperactive neoplasms.

## Results

### MAPK signaling pathway is activated in response to rapamycin treatment

**Fig. 1:**
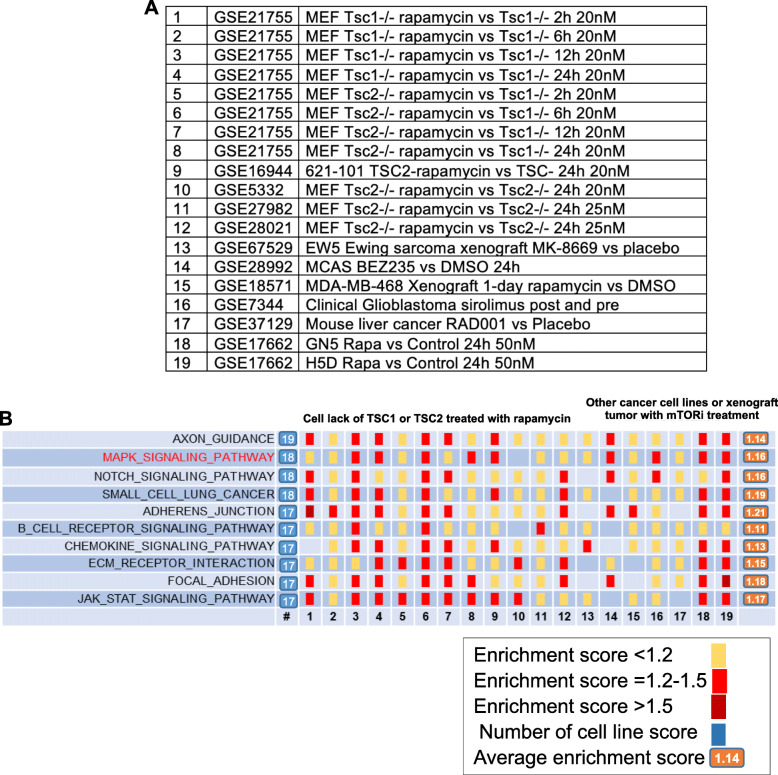
Bioinfomatic analysis of rapamycin enhanced signaling pathway in TSC deficient cells. **a** Publically available gene expression datasets were re-analyzed. **b** Gene set enrichment analysis was performed. Top 10 upregulated signaling pathways in response to rapamycin treatment relative to vehicle-treatment were indicated

To explore the possibility of selectively killing tumor cells with high mTORC1 activity, we performed bioinformatic analysis using various tumor cells including TSC1 and TSC2-deficient cells (GEO accession number GSE16944 [15], GSE21755 [16], GSE5332 [17], GSE27982 [18], GSE28021 [18], GSE67529, GSE28992 [19], GSE18571 [20], GSE7344 [21], GSE37129 [22] and GSE17662 [23]) (Fig. 1a). Gene set enrichment analysis identified top 10 up-regulated signaling pathways in resposne to rapamycin treatment that were conserved in all cell types analysed (Fig. 1b). MAPK signaling pathway is one of the upregulated pathways induced by rapamycin treatment. Other rapamycin-upregulated pathways include axon guidance, notch signaling pathway, small cell lung cancer, adherent junction, B cell receptor signaling pathway, chemokine signaling pathway, ECM receptor interaction, focal adhesion, and JAK/STAT signaling pathway.

### Tsc2-deficient xenograft tumors become refractory to rapamycin treatment

**Fig. 2:**
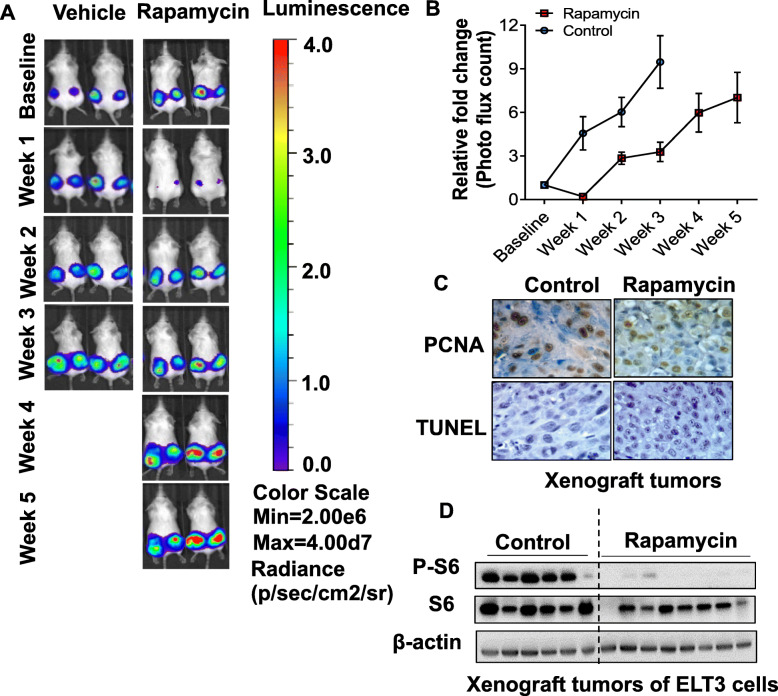
Tsc2-deficient xenograft tumors become refractory to rapamycin treatment. **a** Female CB17-scid mice were inoculated with ELT3-luciferase cells subcutaneously. Mice were treated with either vehicle or rapamycin for 5 weeks. **b** Bioluminescent intensity in xenograft tumors was recorded and quantified weekly. The left Y-axis indicated the relative tumor growth versus the baseline quantification before drug treatment. **c** Immunohistochemistry staining of PCNA and TUNEL. **d** Immunoblotting analysis of phospho-S6 (S235/236) of xenograft tumors

To determine the in vivo efficacy of rapamycin on tumor growth, we first generated xenograft tumors of Tsc2-deficient Eker Rat uterine leiomyoma-derived luciferase-tagged cells [24–26]. The tumor growth was recorded by non-invasive imaging. Rapamycin treatment for one-week resulted in drastic decrease of tumor volume dramatically due to one-week rapamycin treatment. However, tumor rebounded rapidly despite rapamycin treatment was continued for 1 week (Fig. 2a). The tumor growth was monitored for 5 weeks during rapamycin treatment. Interestingly, xenograft tumors persistently progressed from week 2 of the treatment (Fig. 2b). By week 5 of rapamycin treatment, tumors became ulcerated and reached the study endpoints. We performed immunohistochemistry using cell proliferative marker proliferating cell nuclear antigen (PCNA) and found that rapamycin-treated xenograft tumors exhibited high levels of nuclear PCNA staining, comparable to those detected in control tumors (Fig. 2c), indicating that rapamycin does not affect cell proliferative status. To determine the effect of rapamycin on tumor cell death, TUNEL staining was performed in the same set xenograft tumor specimens used for PCNA staining. We did not observe positive TUNEL staining in xenograft tumors of control vehicle-treated or rapamycin-treated mice (Fig. 2c), indicating that rapamyicn does not induce the death of tumor cells. To assess the effect of rapamycin on mTORC1 inhibition in xenograft tumors, we performed immunoblotting analysis and found that S6 phosphorylation was markedly decreased in response to rapamycin treatment in xenograft tumors relative to vehicle control (Fig. 2d). Collectively, our data show that long-term effective inhibition of mTORC1 by rapamycin promotes tumor refractory growth in TSC.

### Rapamycin is cytostatic but not cytotoxic in TSC2-deficient cells in vitro

**Fig. 3:**
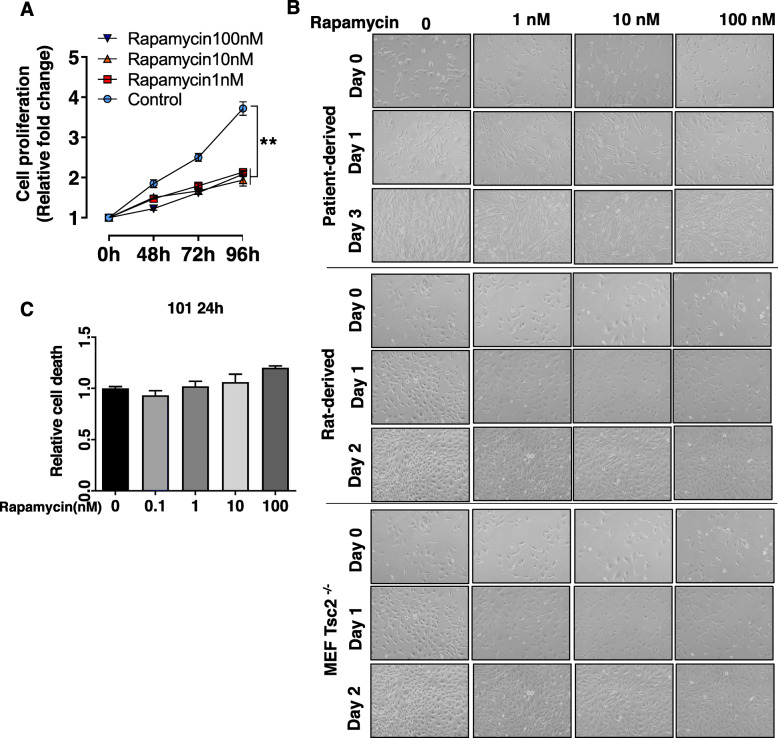
Rapamycin is cytostatic but not cytotoxic in vitro. **a** Cell proliferation of TSC2-deficient patient-derived cells treated with escalating concentrations of rapamycin. **b** Phase contrast microscopy of TSC2-deficient patient-derived, rat uterine leiomyoma-derived, *Tsc2*^−/−^ MEFs and *Tsc1*^−/−^ MEFs treated with rapamycin. **c** Propidium iodide exclusion assay of TSC2-deficient patient-derived cells treated with escalating concentrations of rapamycin as indicated. ** *P* < 0.01, the Student’s t-test

To determine whether rapamycin affects cell proliferation and death in vitro, we use TSC2-deficient patient-derived cells [27, 28], Tsc2-deficient rat uterine leiomyoma-derived cells [24, 25], and *Tsc2*^−/−^*p53*^−/−^ mouse embryonic fibroblasts (MEF) and their TSC2-exressing counterpart controls [29]. Crystal Violet staining showed that rapamycin treatment up to 96 h (1 nM to 100 nM) significantly decreased cell proliferation relative to vehicle control (Fig. 3a). Phase-contrast microscopy showed rapamycin slowed the growth of TSC2-deficient cells without inducing the death of TSC2-deficient patient-derived cells, rat uterine-leiomyoma-derived cells, or *Tsc2*^−/−^ MEFs (Fig. 3b). Furthermore, Propidium iodide exclusion assay showed that rapamycin treatment (1 nM – 100 nM) did not induce the death of TSC2-deficient patient-derived 621–101 cells (Fig. 3c). Collectively, our data demonstrate that rapamycin exhibits cytostatic effect but not cytocidal effect on TSC2-deficient cells in vitro.

### Rapamycin promotes MAPK activation in mTORC1-hyperactive tumor cells in vitro

**Fig. 4:**
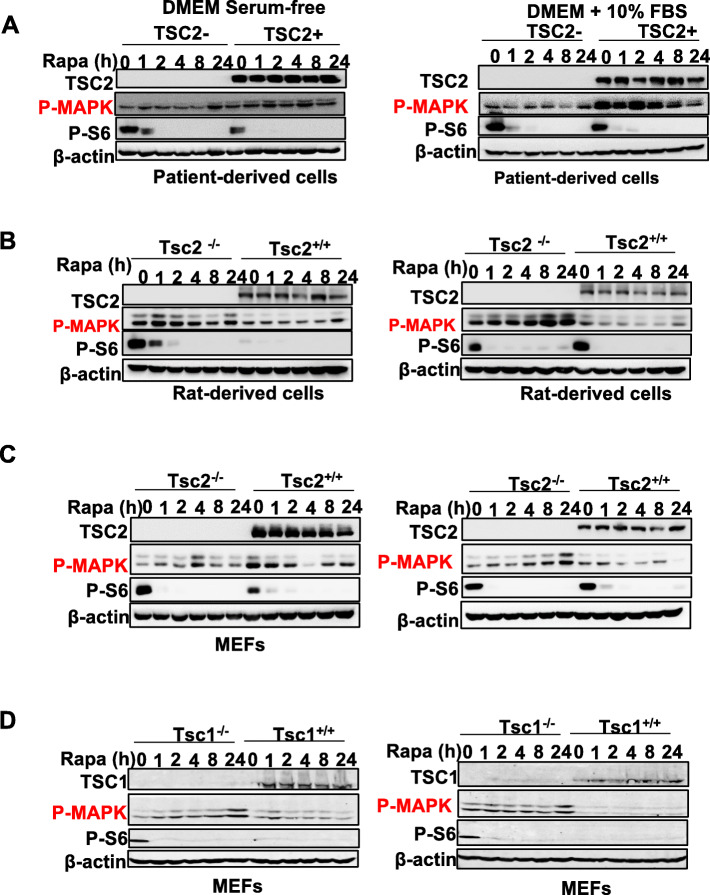
Rapamycin promotes MAPK signaling pathway in TSC2 deficient cells. Immunoblots of TSC2-deficient (**a**) patient-derived cells, **b** rat uterine leiomyoma-derived cell, **c** *Tsc2*^−/−^ MEFs, and **d** *Tsc1*^−/−^ MEFs, treated with rapamycin at indicated time points. Immunoblotting analyses of TSC2, phospho-MAPK (T202/Y204), and phospho-S6 (S235/236) were shown

Our bioinformatic analysis identified activation of MAPK signaling pathway genes in a panel of TSC-deficient cells (Fig. 1). To further assess the MAPK activation, we performed immunoblotting analysis of MAPK phosphorylation in TSC2-deficient patient-derived cells, Tsc2-deficient rat uterine-leiomyoma-derived cells, *Tsc2*^−/−^ mouse embryonic fibroblast (MEFs), and *Tsc1*^−/−^ MEFs, and their TSC2- or TSC1-reexpressing counterparts cultured in nutrient-rich medium containing 10% FBS or nutrient-deprived FBS-free medium, repersenting two basal levels of MAPK phosphorylation. We found that rapamycin selectively promoted MAPK phosphorylation in TSC1- or TSC2-deficient cells but not in TSC1- or TSC2-reexpressing cells (Fig. 4a-d). We also observed that rapamycin treatment decreased S6 phosphorylation as expected.

### Dual inhibition of mTORC1 and MAPK induces the death of TSC2-deficient patient-derived cells in vitro

**Fig. 5:**
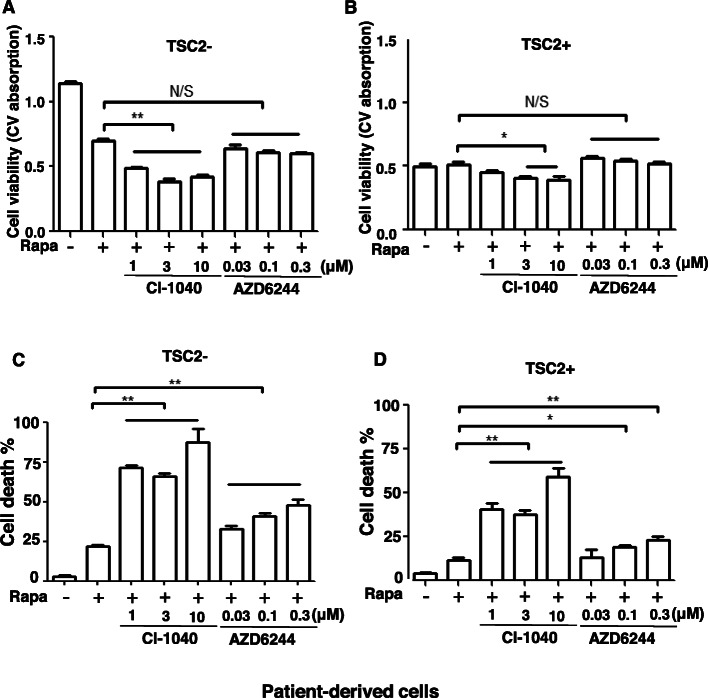
Combinational suppression of mTORC1 and MAPK induces cell death in vitro. TSC2-deficient or TSC2 re-expressing patient-derived cells were treated with vehicle control, rapamycin, or rapamycin combined with AZD6244 or CI-1040. **a**-**b** Cell viability was exmained using crystal violet staining assay (*n* = 8). **c**-**d** Cell death was quantified using Propidum iodine excursion assay (*n* = 8). * *P* < 0.05; ** *P* < 0.01, the Student’s t-test

To test whether dual inhibition of mTORC1 and MAPK synergistically affects cell survival, we first examined cell viability using crystal violet staining. Rapamycin single treatment decreased the viability of TSC2-deficient patient-derived cells (Fig. 5a), but not TSC2-reexpressing cells (Fig. 5b). Importantly, dual treatment of rapamycin and CI-1040, an MEK1/2 inhibitor, significantly decreased the viability of TSC2-deficient cells, and moderately reduced the viability of TSC2-reexpressing patient-derived cells, relative to rapamycin treatment alone (Fig. 5a, b). However, rapamycin plus AZD6244, an MEK1 inhibitor, did not affect the viability of TSC2-deficient or TSC2-reexpressing patient-derived cells (Fig. 5a, b), indicative of a differential effect of MEK1/2 and MEK1 on cell viability in TSC2-deficient patient-derived cells.

To determine the combinatorial effect of rapamycin and MEK1/2 inhibitor on cell survival, we preformed Propidium iodide exclusion assay and found that CI-1040 in combination with rapamycin substantially induced cell death relative to rapamycin treatment in TSC2-deficient and TSC2-reexpressing patient-derived cells (Fig. 5c, d). AZD6244 in combination with rapamycin moderately induced the death of TSC2-deficient and TSC2-reexpresing patient-derived cells (Fig. 5c, d), further indicating the differentially effect of MEK1/2 and MEK1 on the survival of TSC2-deficient patient-derived cells.

## Discussion

The mTORC1 is a serine/threonine protein kinase and plays crucial roles in transcriptional regulation, initiation of protein synthesis, ribosome biogenesis, metabolism, and apoptosis. The deregulation of mTORC1 signaling pathway is frequently observed in cancers and other diseases due to aberrant expression of numerous oncogenes and tumor suppressors [5, 30]. mTORC1 signaling pathway has been the key targets for cancer treatment [31–33]. Although mTORC1 inhibitors have activity in some cancer types, only small population of patients treated with these agents exhibited substantial clinical benefit [34].

mTOR1 pathway is the main therapeutic target for TSC and LAM patients. mTORC1 inhibitors, sirolimus (rapamycin) and everolimus (RAD001), have been approved by FDA for the treatment of TSC-associated subependymal Giant cell astrocytoma in brain (everolimus) [2, 35], renal angiomyolipoma (everolimus) [36], and pulmonary lymphangioleiomyomatosis (sirolimus) [10]. Everolimus has also been approved for the treatment of TSC-associated SEGA and renal angiomyolipoma [37]. Rapamycin (sirolimus) acts by forming complex with the intracellular binding protein FK506-binding protein (FKBP121), such complex in turn binds to the FKBP12-rapamycin binding (FRB) domain of the mTORC1 molecule to inhibit mTORC1 activity [38, 39]. mTORC2 function is intact under acute inhibition, however, it has been noted that long-term rapamycin treatment decreases mTORC2 signaling in primary human dermal microvascular endothelial cells [40] and several cell lines [41]. Everolimus, known as RAD001, is a derivative of sirolimus that acts via similar mechanism [42]. It shares the central macrolide chemical structure with sirolimus, which allows for interaction with FKBP12 [43, 44]. The tissue selectivity of everolimus has also been noted, preferably accumulated in brain mitochondria relative to sirolimus [44].

Currently, there is no single study that directly compares the therapeutic effect of sirolimus and everolimus in TSC management [38, 44, 45]. Clinical decisions are based on clinical trial experiences in the setting of certain TSC manifestations. Sirolimus is generally used to manage TSC-LAM [10], while Everolimus is favored over sirolimus in treating SEGA [44, 46]. Although our current studies focus on the impact of rapamycin on pro-survival of TSC mutant cells, it will be interesting to examine the effect of everolimus on the survival of TSC mutant cells.

Recently, the therapeutic benefit of cannabidiol has been proposed in TSC associated epilepsy [47]. Cannabidiol is a marijuana plant extract that has been studied as an anticonvulsant medicine for treatment-resistant epilepsy with acceptable tolerance [48, 49]. Hess and colleague observed decreased weekly seizure frequency in TSC patients with refractory epilepsy under cannabidiol treatment. In addition to the fact that cannabidiol is yet to be FDA approved, there is no conclusive evidence supporting the effect of cannabidiol exceeding traditional anti-seizure therapy such as the benzodiazepine, GABA analog vigabatrin and ketogenic diet in the management of TSC associated epilepsy [50–53]. Moreover, the specificity of cannabidiol to target the unique mechanism of TSC pathogenesis has not been elucidated.

Preclinical studies including ours have demonstrated the effectiveness of sirolimus, an mTORC1 inhibitor, in multiple animal models of TSC [26, 54–58]. The effect of mTOR inhibitors on TSC tumors in these experiments has been consistently cytostatic rather than cytotoxic, and is variable in efficacy; tumors typically regrow upon the cessation of treatment [59, 60]. Therefore, these preclinical models have become powerful tools in the assessment of potential therapies for TSC. However, the molecular mechanism of the sirolimus-induced cytostatic effect on TSC tumors is not totally elucidated. Our recent study reported that xenograft tumors of Tsc2-deficient rat uterine leiomyoma-derived ELT3 cells became resistant to rapamycin treatment [61]. In this study, we observed that xenograft tumors of ELT3 cells potently responded to rapamycin within 1 week of treatment, however, tumors became refractory from week 2 of rapamycin treatment. This rapamycin resistant growth is consistent with the study by Valianou et al. [61]. In our xenograft tumor study, we used bioluminescent imaging approach to quantify the tumor growth in response to rapamycin treatment, enabling quantification of viable tumor cells in vivo.

Treatment with sirolimus alone has a suppressive rather than remission-inducing effect in majority of tumor models with dysregulated mTORC1 [62, 63]. mTORC1 inhibition leads to upregulation of pro-survival mediators including autophagy and paradoxically increases the growth of Tsc2-null cells [58, 64–66]. Specifically, inhibition of mTORC1 leads to MAPK pathway activation through a PI3K-dependent feedback loop in human cancer [67]. Using bioinformatic approach and immunoblotting analyses, we identified activation of MPAK signaling pathway among other pro-survival pathways in a panel of TSC-deficient cells, and rapid and sustained activation of MAPK in TSC-deficient cells, in agreement with other findings in prostate cancer cells [67].

High-throughput chemical screens in mTORC1-hyperactive patient renal angiomyolipoma-derived and *Tsc2*^−/−^ MEFs cells identified compounds that selectively induce cell death through oxidative stress-dependent mechanisms within 72 h of drug treatment [68, 69]. Thus, there is an unmet need for identifying agents that act with chronic sirolimus treatment to kill mTORC1-hyperactive cells. Our identification of rapamycin-induced MAPK activation prompted us to perform studies of dual inhibition of MAPK and mTORC1 in TSC-deficient cells. We found that MAPK inhibition attenuated rapamycin-induced cytostasis and promoted the death of TSC-deficient cells in vitro.

A potential mechanism by which active-site mTOR or dual inhibitors of PI3K/mTOR promotes MEK1/2-MAPK signaling pathway activation is via enhanced EGFR activity. A recent RNAseq analysis by Valianou et al. identified rapamycin-induced upregulation of EGFR signaling pathway in rapamycin-resistant ELT3 cells [61]. The EGFR tyrosine kinase activity and affinity for its ligands are negatively regulated by protein kinase C (PKCα) via phosphorylation at Thr654 [70]. Studies indicate that mTORC2 mediates PKCα phosphorylation [71, 72]. Interestingly, the mTORC2-dependent phosphorylation of PKCα plays an important role in its maturation, stability, and signaling [71, 72]. It is plausible, therefore, that suppression of mTORC2-mediated post-translational processing of PKCα interferes with negative feedback of PKCα on EGFR, thereby leading to hyperactivation of EGFR and activation of MAPK signaling in response to EGFR agonists or GPCR transactivation [73]. Future studies of the impact of EGFR-mediated MAPK activation on the survival of mTROC1 hyperactive cells will provide novel mechanistic targets for therapeutic application for TSC.

## Conclusions

In the past decade, remarkable progress has been made in demonstrating the efficacy of sirolimus and everolimus in management of TSC and LAM patients. Rapamycin and Rapalogs that target mTOR activity offer an additional value which would help in the treatment of TSC and LAM. However, the effect of sirolimus and everolimus on reducing tumor size or improving symptoms has been consistently cytostatic rather than cytotoxic; tumors typically regrow and symptoms resume upon the cessation of treatment. In this study, we have revealed that mTORC1 inhibition using rapamycin results in a compensatory activation of MAPK in TSC1- and TSC2-deficient cells. This enhanced MAPK signaling pathway was associated with enhanced survival of TSC-deficient cells in vitro. Dual inhibition of mTORC1 and MAPK triggers the death of TSC2-deficient cells. Taken together, our study reveals a novel approach of dual targeting of mTORC1 and MAPK pathways to induce tumor remission in TSC and other mTORC1 hyperactive neoplasms.

## Materials and methods

### Gene set enrichment analysis

Re-analysis of publicly available expression array data (GEO accession number GSE16944 [15], GSE21755 [16], GSE5332 [17], GSE27982 [18], GSE28021 [18], GSE67529, GSE28992 [19], GSE18571 [20], GSE7344 [21], GSE37129 [22] and GSE17662 [23]) was performed using the online tool Gene Pattern (Broad Institute).

### Cell culture and reagents

Cell culture media and supplements were from GIBCO (Frederick, MD). *Tsc2*^−/−^*p53*^−/−^ and *Tsc2*^+/+^*p53*^−/−^ mouse embryonic fibroblasts (MEFs) were developed previously [29]. Mouse expression arrays of *Tsc2*^−/−^*p53*^−/−^ and *Tsc2*^+/+^*p53*^−/−^ MEFs were preformed [16, 18]. An immortalized TSC2-deficient human cell line derived from angiomyolipoma of a LAM patient [28], and its corresponding TSC2-rescued control cell line has been described previously [15]. In brief, patient-derived cells were transfected with pcDNA3.1zeo-hTSC2 or its corresponding empty vector control pcDNA3.1zeo. Stable clones expressing TSC2 were selected using Zeocin for 2 weeks as described previously [74]. Eker rat uterine leiomyoma-derived Tsc2-deficient cells (ELT3) were developed by Howe et al. [24, 25]. ELT3 cells were transduced with a retroviral plasmid pMSCVneo-hTSC2 or its corresponding empty vector pMSCVneo, and then selected with neomycin for 2 weeks. Stable clones were characterized for TSC2 expression [75]. Cells were cultured in DMEM supplemented with 10% fetal bovine serum (FBS), and 1% penicillin-streptomycin-amphotericin B (PSA). Experiments were performed in triplicate for biochemical analyses. Cells were seeded at a density of 2 × 10^5^ cells/ml in 6-well plates in regular growth media for 24 h. Six or 24 h later, cell lysates were prepared using RIPA buffer supplemented with protease inhibitor cocktail (Sigma) and phosphatase inhibitor cocktail (Sigma). Protein concentration was determined using Bradford assay (BioRad Laboratories Inc. Hercules, CA).

### Cell viability assay

Cells were seeded at a density of 5 × 10^4^/ml in a 96-well plate for 24 h and then treated with inhibitors or vehicle control for 24 h. Cell numbers were quantified using CyQuant (Invitrogen, Carlsbad, CA) or crystal violet staining assay. Values are expressed as mean ± SEM; *n* = 8/group.

### Animal studies

The University of Cincinnati Institutional Animal Care and Use Committee approved all procedures described according to standards as outlined in The Guide for the Care and Use of Laboratory Animals. For xenograft tumor study, 2 × 10^6^ ERL4-luciferase-tagged (TSC2-null) cells were inoculated bilaterally into the posterior back region of female intact CB17-SCID mice (Taconic) as previously described [26, 76]. For the current study, 9–10 week-old CB-17 SCID mice were treated with vehicle control or 2 mg/kg rapamycin (dissolve in 0.25% Tween 80, 0.25% polyethylene glycol 400, i.p.) every day for 3 weeks. The tumors were harvested 3 weeks post cell inoculation. Tumor growth were monitored weekly using a non-invasive imaging by IVIS (Perkin Elmer). All efforts were made to reduce suffering of the animals and minimize the number of animals used in the study.

### Bioluminescent reporter imaging

Ten minutes before imaging, animals were injected with luciferin (Xenogen) (120 mg/kg, i.p.). Bioluminescent signals were recorded using the Xenogen IVIS System. The total photon flux of tumors was analyzed [26].

### Immunohistochemistry

Immunohistochemistry (IHC) was performed on paraffin-embedded 10 μm-sections. Slides were deparaffinized, and antigen retrieval was performed using Dako Target Retrieval Solution pH 6 (Dako, Carpinteria, CA). Sections were stained by the immunoperoxidase technique using DAB substrate (Dako EnVision System HRP) and counterstaining with hematoxylin. After staining, slides were viewed on a Nikon Eclipse E400 microscope, and images captured using Spot Insight digital camera with Spot software (Diagnostic Instruments, Sterling Heights, MI).

### Western blotting

Protein samples were analyzed by SDS-PAGE using 4–12% NuPAGE Gel (Invitrogen, Carlsbad, CA), and transferred to a nitrocellulose membrane. Immunoblotting was performed by standard methods using HRP-conjugated secondary antibodies, and chemiluminescence using Supersignal West Pico Chemiluminescent substrate (Thermo Scientific) and exposure using Syngene G:Box. All antibodies were purchased from Cell Signaling (Danvers, MA).

### Statistical analyses

All data are shown as the mean ± S.E.M. Measurements at single time points were analyzed by ANOVA and then using a two-tailed t-test (Student’s t test). Time courses were analyzed by repeated measurements (mixed model) ANOVA and Bonferroni post-t-tests. All statistical tests were performed using GraphPad Prism 5.0 (GraphPad Software, San Diego, CA, USA) and *p* < 0.05 indicated statistical significance.

## Data Availability

All data generated or analyzed during this study are included in this published article.

## Abbreviations

ELT3: Eker rat uterine leiomyoma-derived cells
ERL4: ELT3 cells expressing luciferase
LAM: Lymphangioleiomyomatosis
MAPK: Mitogen-activated protein kinase
mTOR: Mechanistic target of rapamycin
PCNA: Proliferating cell nuclear antigen
PS6: Phospho-ribosomal protein S6 (Ser235/236)
RAPTOR: Regulatory-associated protein of mTOR
Rheb: Ras homologue enriched in brain
RICTOR: Rapamycin-insensitive companion of mTOR
S6K: Ribosomal protein S6 kinase
TSC: Tuberous Sclerosis Complex
4E-BP1: Translational repressor 4E-binding protein 1
